# Monitoring the Behavior of Na Ions and Solid Electrolyte Interphase Formation at an Aluminum/Ionic Liquid Electrode/Electrolyte Interface via Operando Electrochemical X-ray Photoelectron Spectroscopy

**DOI:** 10.1021/acsami.4c02241

**Published:** 2024-06-27

**Authors:** Roxy Lee, Tim S. Nunney, Mark Isaacs, Robert G. Palgrave, Avishek Dey

**Affiliations:** † Department of Chemistry, University College London, 20 Gordon Street, London WC1H 0AJ, U.K.; ‡ Thermo Fisher Scientific, Unit 1, The Felbridge Centre, East Grinstead, West Sussex RH19 1XP, U.K.; § HarwellXPS, Research Complex at Harwell, Rutherford Appleton Lab, Didcot OX11 0FA, U.K.; ∥ The Faraday Institution, Quad One, Harwell Science and Innovation Campus, OX11 0RA Didcot, U.K.

**Keywords:** XPS, operando, cyclic voltammetry, SEI, sodium ion, ionic liquid

## Abstract

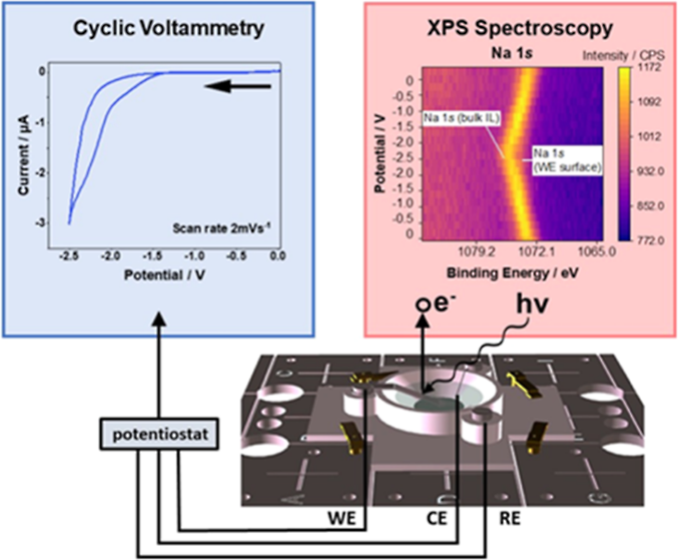

In electrochemical energy storage devices, the interface between the electrode and the electrolyte plays a crucial role. A solid electrolyte interphase (SEI) is formed on the electrode surface due to spontaneous decomposition of the electrolyte, which in turn controls the dynamics of ion migration during charge and discharge cycles. However, the dynamic nature of the SEI means that its chemical structure evolves over time and as a function of the applied bias; thus, a true *operando* study is extremely valuable. X-ray photoelectron spectroscopy (XPS) is a widely used technique to understand the surface electronic and chemical properties, but the use of ultrahigh vacuum in standard instruments is a major hurdle for their utilization in measuring wet electrochemical processes. Herein, we introduce a 3-electrode electrochemical cell to probe the behavior of Na ions and the formation of SEI at the interface of an ionic liquid (IL) electrolyte and an aluminum electrode under *operando* conditions. A system containing 0.5 molar NaTFSI dissolved in the IL [BMIM][TFSI] was investigated using an Al working electrode and Pt counter and reference electrodes. By optimizing the scan rate of both XPS and cyclic voltammetry (CV) techniques, we captured the formation and evolution of SEI chemistry using real-time spectra acquisition techniques. A CV scan rate of 2 mVs^–1^ was coupled with XPS snapshot spectra collected at 10 s per core level. The technique demonstrated here provides a platform for the chemical analysis of materials beyond batteries.

## 1 Introduction

As a major contender for future renewable energy storage, Na-ion batteries (NIBs) offer lower cost and sustainability compared to their Li-ion counterparts.^1−3^ There is therefore an urgent need to develop NIBs for grid-scale applications, and substantial advancements have been made in the development of high-capacity and high-voltage electrode materials for high-energy-density NIBs.^4−7^ However, progress is hindered by inferior reversibility arising from the severe instability of the solid electrolyte interphase (SEI) and uncontrollable side reactions at the electrode/electrolyte interface.^8,9^ The SEI, which ideally is ionically conducting and electrochemically stable, is formed at the electrode/electrolyte interface during the first charge/discharge cycle of the battery. The characteristics of this SEI layer eventually govern the battery performance and lifetime; thus, an improved understanding of the interfacial chemistry is crucial to obtain high-energy-density batteries.

A number of in situ techniques have been used to characterize the SEI, including neutron reflectometry and atomic force microscopy,^10^ soft X-ray absorption spectroscopy,^11^ Raman spectroscopy,^12^ transmission electron microscopy,^13−15^ scanning electron microscopy (SEM),^16^ and Fourier transform infrared spectroscopy.^17^ An emerging area with great prospects is that of in situ electrochemical X-ray photoelectron spectroscopy (XPS).^18^ XPS is a powerful surface analysis technique with which a wealth of information from the top ca. ∼5 nm of a sample can be obtained, including identification and quantification of elemental composition and determination of oxidation state and local chemical environment.^19^ Although XPS has extensively been used for ex situ post-mortem analysis of the battery components,^20−22^ such studies do not always reflect the same properties as those under operating conditions. A standard ex situ technique involves cycling the battery using a defined protocol, after which the components are separated and characterized. However, these ex situ measurements fail to provide information about the dynamic chemical evolution of the SEI layer. The information thus represents snapshots at a particular point of the electrochemical cycle, considering that the effect on SEI chemistry during sample transfer under different conditions/atmospheres is negligible.

The requirement for ultrahigh vacuum (UHV) is one of the major challenges to in situ electrochemical XPS; therefore, some researchers have turned to near-ambient pressure (NAP)-XPS techniques to study liquid electrode/electrolyte interfaces. Favaro et al. used AP-XPS and the dip-and-pull method to probe the electric double layer (EDL) at a solid/liquid electrode/electrolyte interface for a system of pyrazine molecules in water solvent.^23^ An approach to investigate electrochemical studies using UHV XPS systems is to use a graphene window to create a barrier between the liquid and the vacuum environments. Shalom et al. used a microelectrochemical cell sealed with a graphene window to monitor the behavior of Cu species in an aqueous electrolyte under electrochemical control.^24^

Alternatively, the challenge of UHV can be overcome by using ionic liquid (IL) electrolytes. ILs are composed solely of ions and exhibit several desirable properties including high electrical conductivity (up to 100 mS cm^–1^), a wide electrochemical window (up to 5.8 V), and an extended liquid-state temperature range (ca. 173–523 K).^25^ Alongside these beneficial electrochemical properties, the nonflammable and nonvolatile nature makes ILs safer alternatives to organic electrolytes in rechargeable NIBs.^26−28^

Several studies have taken advantage of these unique properties of ILs and used them to study the electrochemical behavior of systems using in situ electrochemical XPS. These include the work of Morales-Ugarte et al., who coupled electrochemical impedance spectroscopy and XPS to study the aging of lithium metal surfaces in IL electrolytes,^29^ and Benayad et al., who studied the evolution of SEI at the boundary between a lithium electrode and IL electrolyte using XPS.^30^ Using in situ electrochemical XPS, Liu et al. monitored the electrochemical behavior of anionic gold species in an IL,^31^ Wibowo et al. studied the electrodeposition of rubidium metal in competition with solvent breakdown,^32^ and Weingarth et al. studied the Pt/[EMIM][BF_4_] system.^33^ Morey et al. utilized operando AES/XPS to study the dynamics of Li plating in solid-state batteries using an electron beam.^34^ However, to our knowledge, there have so far been no *operando* electrochemical XPS studies on Na ion behavior. Recently, ILs have been modified to improve their physiochemical and electrochemical properties. For operando studies under UHV conditions, ILs that have low vapor pressure are critical. We considered 1-butyl-3-methylimidazolium bis(trifluoromethyl sulfonyl)imide (BMIM TFSI), which has been widely studied as an electrolyte. Moreover, BMIM TFSI demonstrated a stable potential window under UHV conditions.

For *operando* measurements, it is important to match the time scales of XPS data acquisition with the electrochemical measurement. Also, it is important to negate the effect of open-circuit potential causing differential peak shifts and fictitious spectral features during XPS data acquisition. To overcome this, the evolution of SEI was studied using a classical 3-electrode configuration. The advantage of a 3-electrode system is the ability to monitor both the potential and current independently and correlate that with the surface chemistry at the electrode–electrolyte interface. In this study, the operando measurements were carried out on an Al electrode. Using aluminum as the anode current collector is one of the viable options to reduce the cost of NIBs since sodium ions do not electrochemically alloy with aluminum as opposed to lithium ions. Also, to avoid an additional carbon contribution to the XPS signal, carbonaceous anode materials like hard carbon were avoided in this study. The focus of this study is to synchronize the techniques of electrochemistry and photoemission spectroscopy in a standard lab-based XPS. To achieve this, we utilized real-time data acquisition to continuously map the changes in surface chemistry during the electrochemical cycling of a Na ion salt in an IL electrolyte.

## 2 Experimental Details

### 2.1 Materials and Synthetic Methods

Methyl imidazole, butyl bromide, and LiTFSI were obtained from Sigma-Aldrich. NaTFSI was purchased from SOLVIONIC, France. Al foils and Pt wires were acquired from Goodfellow, UK. The cell basin was 3D-printed from standard resin using a Form 3+ 3D printer from Formlabs. LiTFSI and NaTFSI were dried in a vacuum oven overnight at 60 °C before use. IL BMIM TFSI was synthesized and purified using the following steps. First, methyl imidazole (0.1 equiv) and 1-bromobutane (0.12 equiv) were stirred at 60 °C under reflux until 1-methylimidazole had completely reacted. The formation of BMIMBr was confirmed using ^1^H NMR. BMIM TFSI was synthesized by metathesis of equimolar BMIMBr and LiTFSI. Both BMIMBr and LiTFSI were dissolved in high-purity DI water and stirred at 60 °C under reflux. After 12 h, there was a complete separation of the IL and water. The IL was then washed with DI water several times and checked with Ag(NO)_3_ until no bromide impurities (AgBr precipitate) could be seen. The IL was then mixed with acetonitrile and activated charcoal, followed by filtration (5 times). Acetonitrile was removed using a rotary evaporator at 60 °C. The electrolyte was prepared by dissolving 0.5 M NaTFSI in 5 mL of IL. Finally, the electrolyte was dried in a vacuum oven at 75 °C for 48 h.

### 2.2 Instrument Details

Operando XPS measurements were made using a Thermo Scientific Nexsa G2 spectrometer (Thermo Fisher Scientific, Brno, Czechia). The electrochemical cell, detailed in the section below, was mounted onto the sample carrier with three connections for applying the bias from the potentiostat (PalmSens4, The Netherlands) connected to the external voltage input on the instrument. Spectra for the operando experiments were collected using a 400 μm X-ray spot and the snapshot spectral acquisition mode, which collects a spectrum across an energy range of ∼20 eV in a single frame, by fixing the analyzer pass energy to 150 eV and utilizing a one-dimensional, signature-corrected detector to collect each point in the spectrum simultaneously. For these experiments, a frame acquisition time of 1s was used, and 10 frames were averaged together. For the line scan experiments used to confirm the position of the boundary of the IL, scanned acquisition mode was used to collect a survey scan with the following acquisition parameters: 200 eV pass energy, 1 eV step size, and 10 ms dwell time. As the cell components were conducting, the dual-beam flood source was not used for data acquisition.

Ex situ XPS analysis was performed using a Thermo NEXSA X-ray photoelectron spectrometer fitted with a monochromated Al kα X-ray source (1486.7 eV), a spherical sector analyzer, and 3 multichannel resistive plate, 128 channel delay line detectors. All data were recorded at 19.2 W and an X-ray beam size of 400 × 200 μm. Survey scans were recorded at a pass energy of 160 eV, and high-resolution scans were recorded at a pass energy of 40 eV. Electronic charge neutralization was achieved using a dual-beam low-energy electron/ion source (Thermo Scientific FG-03). Ion gun current = 150 μA. Ion gun voltage = 45 V. All sample data were recorded at a pressure below 10^–8^ mbar and a room temperature of 294 K. Data were analyzed using CasaXPS v2.3.19PR1.0. Peaks were fitted with a Shirley background prior to the component analysis.

## 3 Cell Design and Data Acquisition

**Figure 1:**
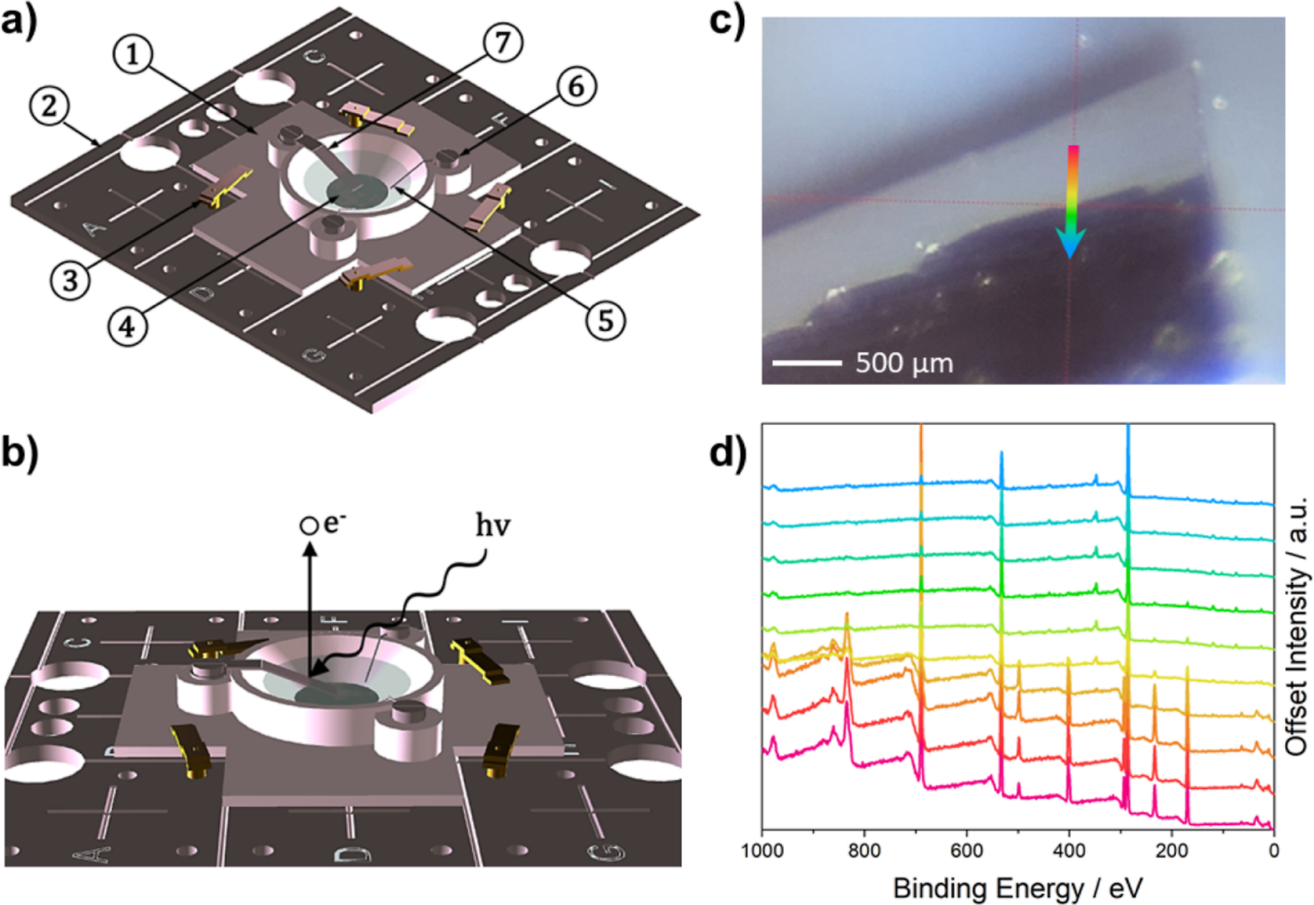
(a) 3D schematic of the in situ XPS cell design; features **1**–**7** are detailed in the text. (b) The cell is designed to enhance the signal from the electrode/electrolyte boundary. The direction of the electrons that will be captured by the detector is indicated. (c) Direction of the line scan to locate the interface; (d) series of survey spectra recorded along the line scan in (c).

The *operando* XPS cell was designed and 3D-printed using AutoCAD 3D software.^35^ A 3D schematic of the cell is detailed in Figure 1a with key features labeled **1–7**. The 3D-printed cell (**1**) is designed to fit the stage (**2**) for the Thermo Scientific Nexsa G2 spectrometer and can be easily fastened by using the standard stage clips (**3**). The basin is filled with an IL electrolyte (**4**) in contact with a wire reference electrode (RE) (**5**), which is fastened in place by a screw (**6**) connected to the bias contacts within the spectrometer (not shown for the sake of clarity). The counter electrode (CE) has an identical setup to the RE, while the working electrode (WE) (**7**) is optimally positioned for electrochemical XPS measurements. In the present work, measurements were performed using an Al WE and Pt wire for both the RE and CE. The electrode/electrolyte interface at the WE is of significant interest, and therefore the design exhibits several features to enable and enhance the signal from this key region. As the position of the detector in the spectrometer and the angle of incident X-rays are fixed, the WE is positioned to maximize the number of ejected photoelectrons reaching the detector. The shallow angle of the basin ensures that incoming X-rays may be focused on the interface and that the WE is submerged in the IL electrolyte at a shallow angle (<45°) to allow ejected photoelectrons to escape the cell and reach the detector. The angle of incoming X-rays focused on the boundary and ejected photoelectrons that will reach the detector is shown schematically in Figure 1b. The procedure to focus the X-ray beam at the interface involves collecting XPS survey spectra for a line scan that spans the boundary between the WE and the IL (Figure 1c). The interface may then be identified by the position at which strong signals can be seen from both the electrode and electrolyte (Figure 1d). Figure S3 shows the line scan taken across the interface.

**Figure 2:**
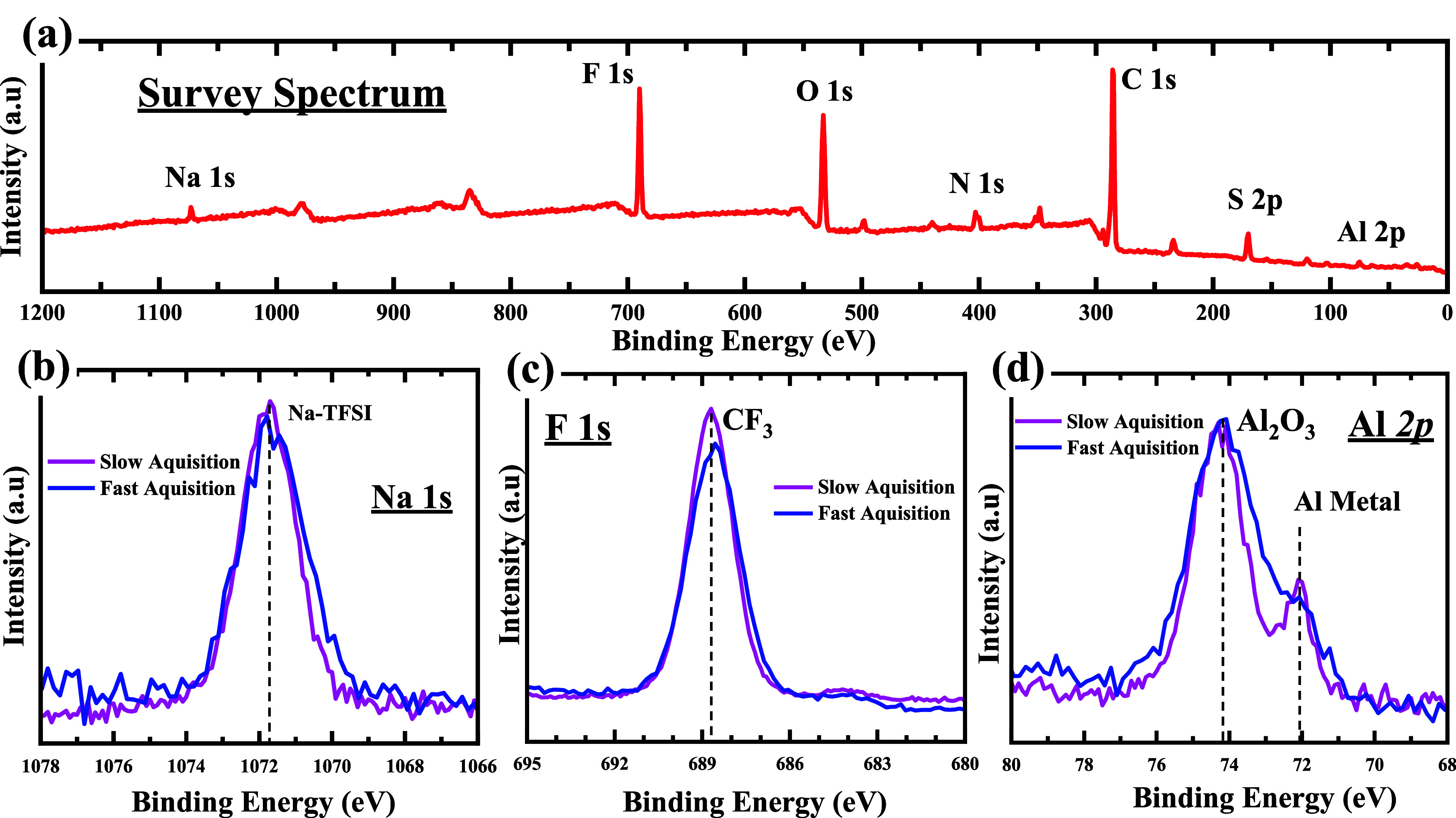
XPS spectra at the Al electrode and IL electrolyte interface acquired at different scan speeds. (a) XPS survey spectrum of the interface. (b–d) Na 1s, F 1s, and Al 2p core-level spectra of the interface.

The acquisition speed of both XPS and cyclic voltammetry (CV) techniques was optimized to perform operando measurements. On one hand, the energy resolution of the XPS spectra is influenced by the pass energy of the acquisition.^36^ This in turn affects the time resolution of the instrument, i.e., the time required to acquire a single spectra with a given signal-to-noise ratio. On the other hand, faster acquisition requires high electron throughput going to the detector, which reduces the energy resolution of the measurement. This type of snapshot scanning mode is available for many lab-based UHV XPS systems. For this measurement, the acquisition parameters of the photoemission spectra were optimized with respect to the scan speed of CV. Figure 2b–d compares the XPS spectra taken under standard, scanned measurement conditions with the snapshot spectra, which achieved fast acquisition (10 s). With fast acquisition, the intensities (electron counts) were similar, but the energy resolution was reduced due to the need to set the spectrometer pass energy to a higher value to give sufficient energy dispersion across the detector. This can be seen from the increased full width of the peaks in the spectra. Most importantly, the two data sets contain the same chemical-state information, as evidenced by the two Al environments (metallic and native oxide) in the Al 2p spectra. The CV scan rate of 2 mVs^–1^ and XPS snapshot scan time of 10 s lead to a variation of 0.02 V during the acquisition of each spectrum, which is expected to cause negligible broadening due to the potential variation. The optimized acquisition times facilitate the collection of XPS spectra that reflect real-time electrochemical processes, and the CV scan rate could be further reduced to investigate the effect this may have on SEI features.

## 4 Results and Discussion

**Figure 3:**
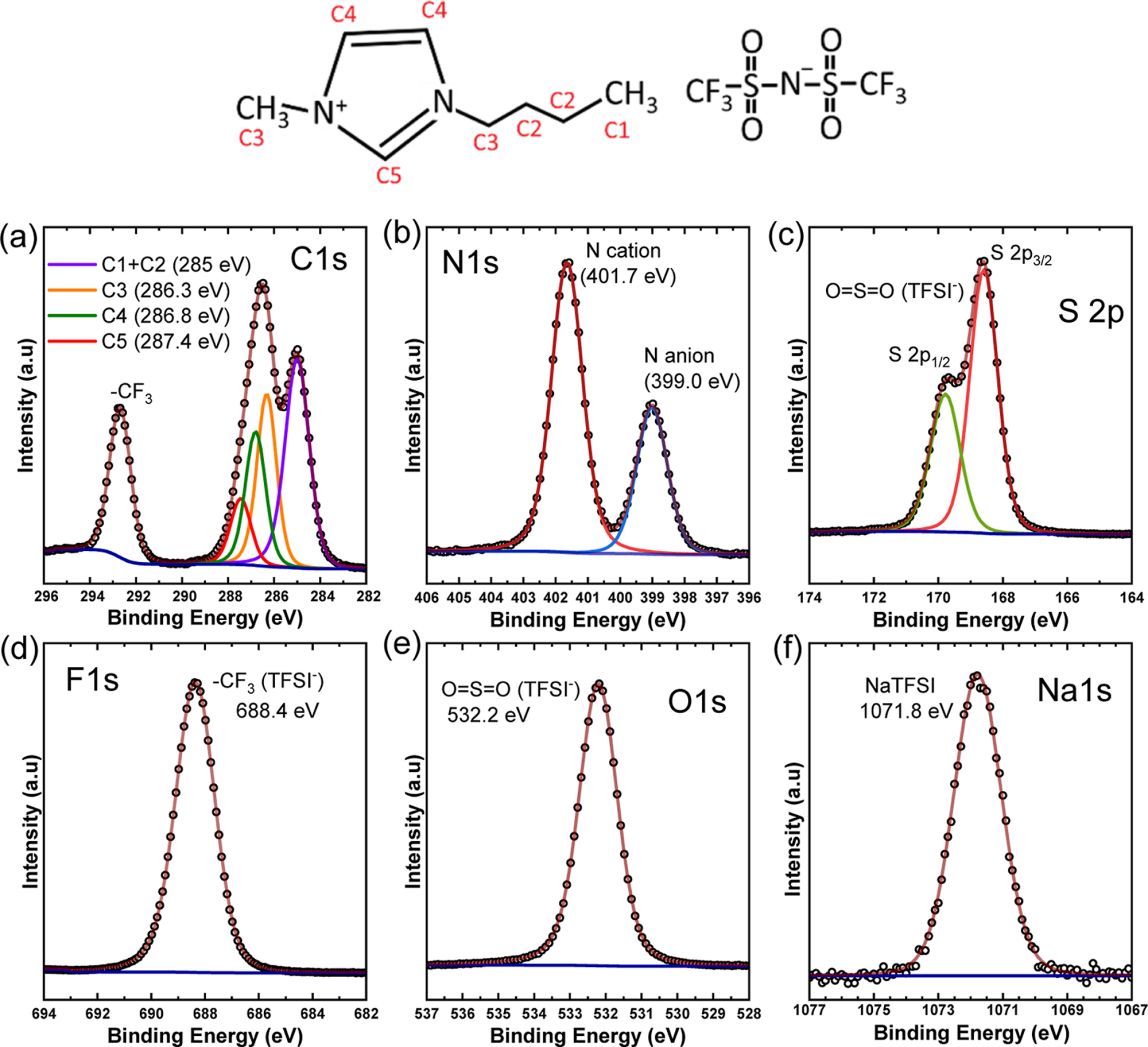
Structure of BMIM TFSI and the corresponding chemical environments of carbon. (a–f) C 1s, N 1s, S 2p, F 1s, O 1s, and Na 1s XPS spectra of BMIM TFSI with 0.5 M NaTFSI.

The system used for this study was 0.5 M NaTFSI dissolved in BMIM TFSI. This IL electrolyte was first analyzed using standard XPS measurements. Figure 3a–f shows the deconvoluted core-level spectra of the constituent elements. Sulfur, fluorine, oxygen, and sodium are seen as being in a single chemical environment in the TFSI^–^ anion and the NaTFSI salt. The nitrogen spectra contain contributions from the anion and cation. The low binding energy (BE) peak at 399 ± 0.2 eV corresponds to the anion, while the intense peak at 401.6 ± 0.2 eV corresponds to the two nitrogen atoms in the imidazole ring. Similar to the nitrogen spectra, the C 1s spectra contain contributions from both the cation and anion. The corresponding carbon environments are highlighted in red on the BMIM cation in Figure 3. The absence of any secondary peak in the spectra is indicative of the purity of the IL electrolyte and the absence of any fluoride or oxide intermediates. The anion has a single carbon environment (−CF_3_), which corresponds to the peak at 292.6 ± 0.2 eV in the C 1s spectra, while the cation contributes to a broad peak between 282 and 290 eV, which can be deconvoluted to five distinct carbon environments (C1, C2, C3, C4, and C5). Here, C1 and C2 are aliphatic carbon, C3 corresponds to carbon bonded to nitrogen in the imidazolium ring, and both C4 and C5 correspond to the carbon in the imidazolium ring. The assignment of the chemical environments that contributed to the deconvoluted C 1s spectra was carried out according to the previous reports.^37−40^ The peak at 285.0 ± 0.2 eV corresponds to the contribution from C1 and C2 aliphatic carbon. The other three components between 286 and 288 eV correspond to C3 (286.3 ± 0.2 eV), C4 (286.8 ± 0.2 eV), and C5 (287.4 ± 0.2 eV) environments, respectively. The fwhm of C3, C4, and C5 were fixed in accordance with the fwhm of the CF_3_ environment (1.0 eV). To accommodate the core-hole broadening of the aliphatic carbon, the fwhm of C1 + C2 was set 0.2 eV larger, i.e., 1.2 eV. From the chemical structure, the stoichiometric ratio of the cation components equates to 1:2:2:3 (C5/C4/C3/C1 + C2). However, the shakeup/off phenomena are known to influence the photoelectrons emanating from the imidazolium ring. To account for this, the stoichiometric contributions corresponding to the C4 and C5 components were reduced by 20%.^38^ The relative peak area ratio for the four cationic components is thus 0.8:1.6:2:3 (C5/C4/C3/C1 + C2).

Ultraviolet photoemission spectroscopy (UPS) using He II (40.8 eV) and He I (22.1 eV) sources was used to probe the valence band structure of the IL and the IL electrolyte. The addition of NaTFSI to BMIM TFSI does not affect the valence band structure of the He II UPS spectra in Figure S1a. However, the addition of NaTFSI reduced the work function of the IL by 0.3 eV. To calculate the work function, both the IL and the IL electrolyte were biased to −9 V and referenced with respect to the Fermi edge of Pt foil. The samples were biased to take the work function samples beyond the instrument work function. The secondary electron cutoff corresponding to He1 excitation for BMIM TFSI was measured to be 16.3 eV, which shifted to 16.6 eV after adding the NaTFSI salt (Figure S1b). Getting a measurement of the work function of the electrolyte is important. The work function of the electrolyte dictates electron transfer at the electrode–electrolyte interface. It has been reported before that without a favorable alignment between the HOMO level of the electrolyte and the electrode, the interface could become too resistive, resulting in the decomposition of the electrolyte and a thicker SEI.^41^

**Figure 4:**
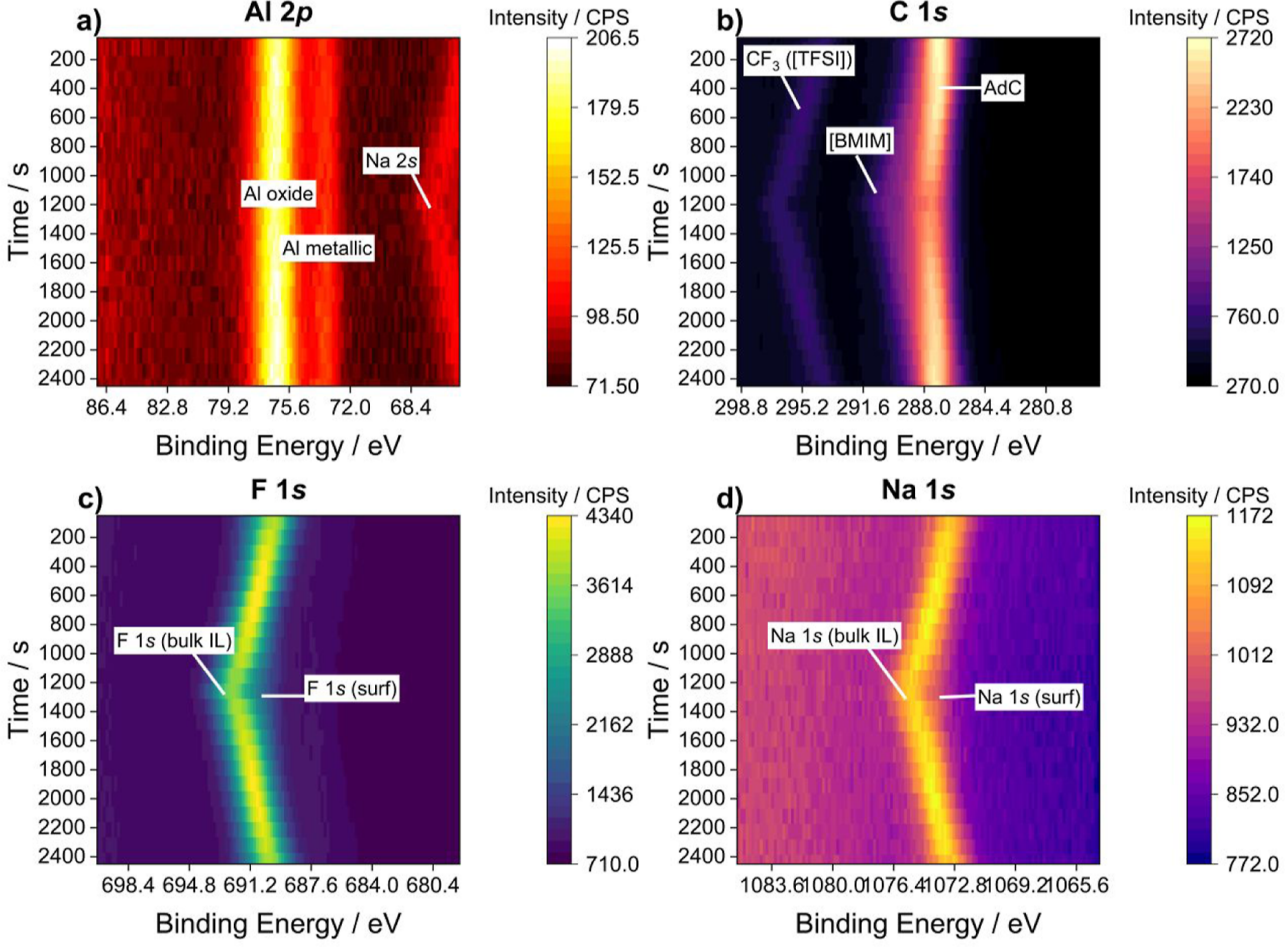
Dynamic XPS spectra taken at the WE/IL interface for (a) Al 2p, (b) C 1s, (c) F 1s, and (d) Na 1s core-level spectra during CV measurements from 0 to −2.5 V.

Turning to the *operando* electrochemical XPS results, the dynamic core-level XPS BE shifts can be visualized by the spectral intensity of the measured BEs as the CV scan time progresses (Figure 4). It is clear that individual species experience different BE shifts due to the applied potential at the WE, which is swept from 0 to −2.5 V and then back to 0 V. Since the WE is grounded in the potentiostatic arrangement, the metallic Al 2p and the native oxide Al_2_O_3_ signals do not shift due to the applied potential, as can be seen by the constant BEs during CV in Figure 4a. This observation is consistent with a previous study by Weingarth et al., where the BEs of electrode species and species in electrical contact with the electrode experienced no shift with the applied potential, whereas species in the bulk electrolyte experienced a roughly ∼−1.0 eV/V shift.^33^ Species in the bulk electrolyte are expected to experience the full potential drop and thus a precise −1.0 eV/V shift, whereas any deviation from this can be expected from species in direct contact with the electrode or within the EDL, where the potential is different.^31,33,42^ Thus, the grounded WE configuration can be used to distinguish between signals arising from species in the electrolyte and the surface of the electrode.

**Figure 5:**
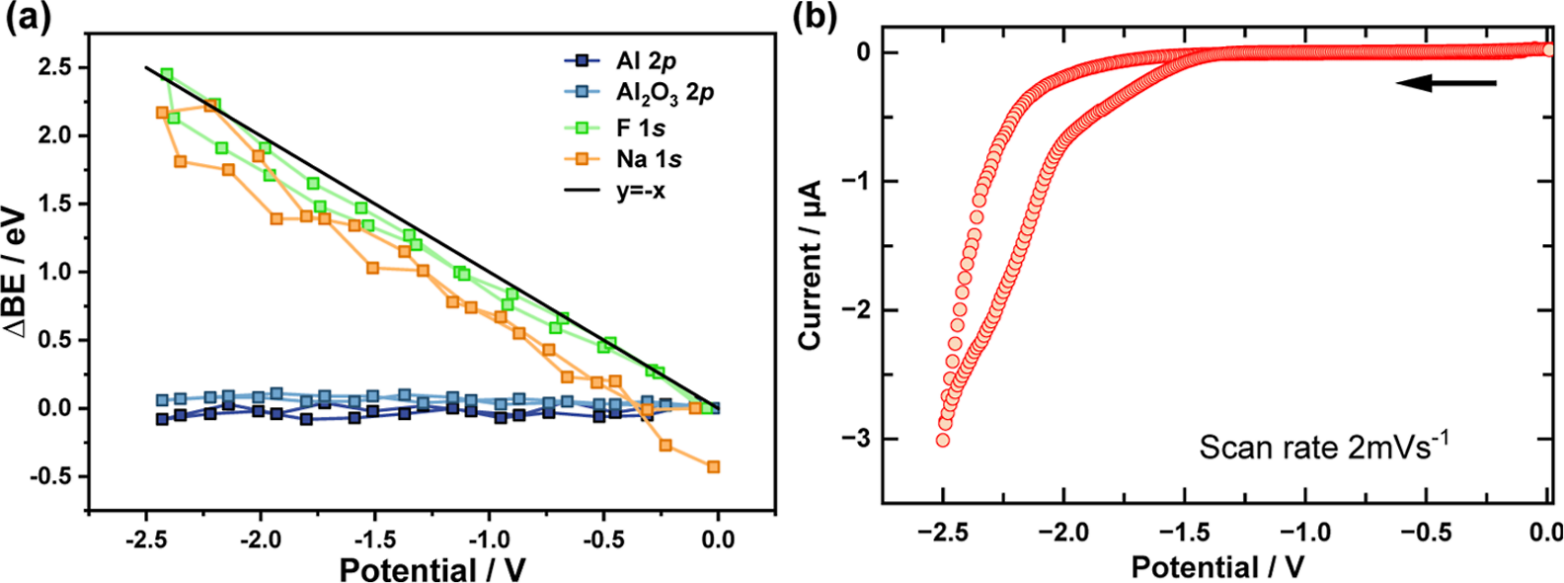
(a) Changes in the BE of the core-level spectra during the (b) operando CV measurement from 0 to −2.5 V. The CV scan rate was 2 mVs^–1^ with the scan direction indicated by the black arrow.

Tracing the shifts of the spectral features for the Al 2p, C 1s, F 1s, and Na 1s spectra during CV reveals signals arising from both the electrode surface and the electrolyte. As discussed above, the Al 2p signals do not shift; however, a faint contribution originating from Na 2s electrons at the low BE edge of the region is seen to shift with the applied potential. The shifts in the C 1s region are complicated by the presence of adventitious carbon at the surface of the electrode, which overlaps with the [BMIM] cation signal. Tracing the signals of the spectral maxima of the Na 1s and F 1s signals reveals a roughly ∼−1.0 eV/V shift (Figure 5a), indicating that the major species in the analysis region are behaving as if they are dissolved in or originate from the bulk electrolyte. The Na 1s *spectral maximum* shifts later than that of F 1s and does not return to the original BE, indicating an irreversible change in the Na 1s spectrum. Inspection of the *operando* CV (Figure 5b) reveals small reduction currents in the potential range of −2.1 to −2.4 V and a steep increase in current around −2.5 V corresponding to decomposition of the electrolyte. The absence of reoxidation peaks is in agreement with the irreversible changes observed in the *operando* XPS spectra.

**Figure 6:**
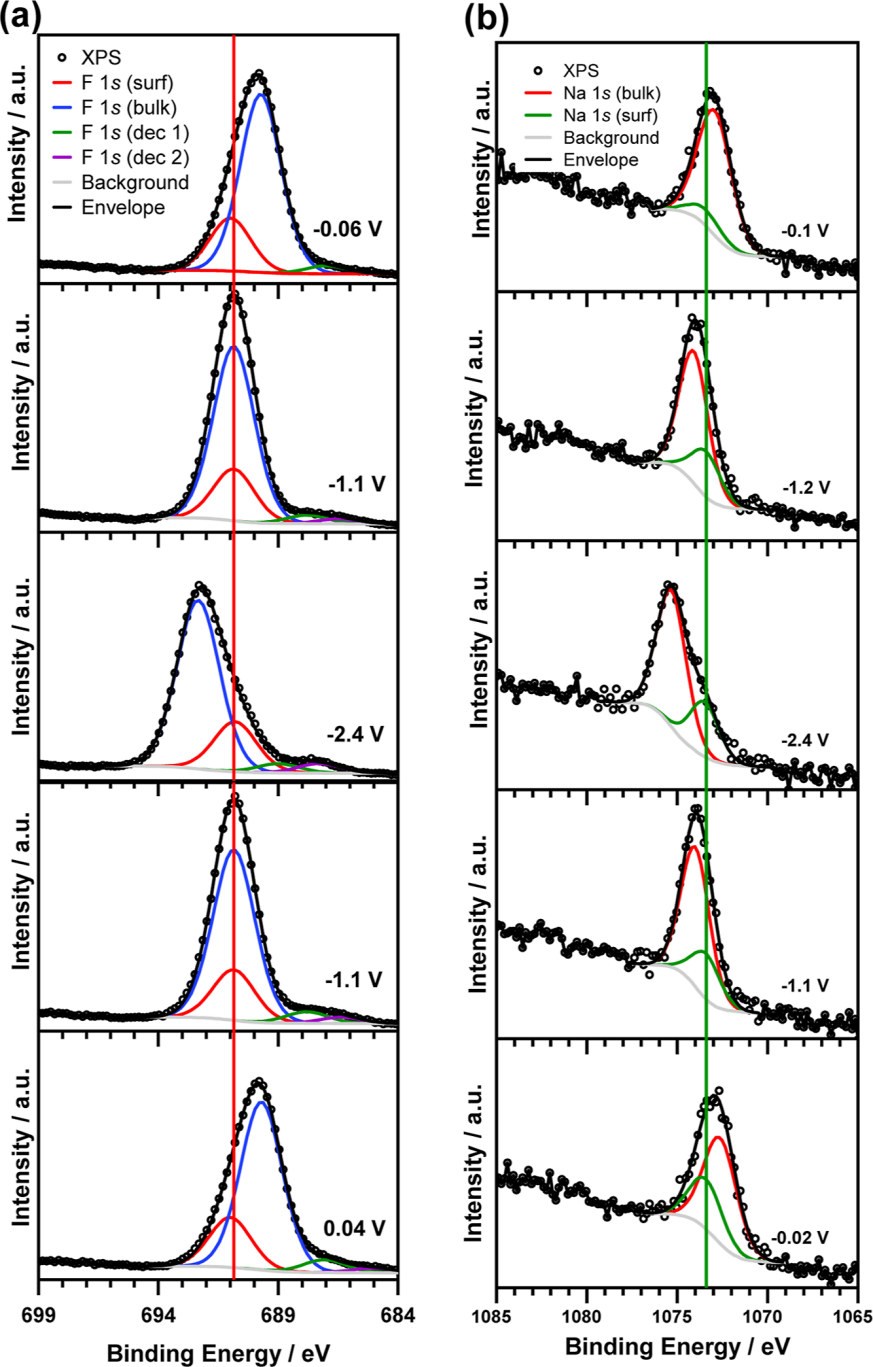
Operando XPS spectra recorded during CV measurements from 0 to −2.5 V showing the (a) F 1s and (b) Na 1s spectra at different potentials. The vertical lines are added as a guide to the eye, highlighting species that do not shift due to the applied potential, which are located at the electrode surface and are labeled “surf”. Species located in the bulk IL are labeled “bulk”, and additional decomposition product peaks in the F 1s spectra are labeled “dec 1” and “dec 2”. The CV scan direction is from the top to the bottom of the figure.

To understand the behavior of species at the surface of the electrode, *operando* F 1s and Na 1s XPS spectra were deconvoluted to model the species at the electrode and in the electrolyte (Figure 6). Note that this is the same data set as displayed in Figure 4, and the displayed spectra are selected to display a range of different applied voltages during CV, with the full set of raw spectra plotted in Figure S2. The Na 1s spectra in Figure 6 are fitted with one component for the electrolyte (labeled “Na 1s bulk”) and one component for Na species at the surface of the electrode (labeled “Na 1s surf”). During CV measurement, the intensity of the species at the electrode surface increases, which is attributed to the formation of Na species in the SEI, and this explains the irreversible shift of the spectral maximum due to the increased intensity of this component. Similarly, in the F 1s spectra, the components in the main peak represent species found at the electrode surface (labeled “F 1s surf”) and species in the electrolyte (labeled “F 1s bulk”). A small component (labeled “F 1s dec 1”) is expected to be a decomposition product dissolved in the electrolyte as this moves similarly to the IL peak during cycling. The IL and salt may already have undergone some decomposition before the *operando* CV during preliminary measurements made to test electrode connections, causing the appearance of surface and bulk decomposition species. During cycling, the appearance of another peak (labeled “F 1s dec 2”) is attributed to further decomposition products, which are also expected to be dissolved in the IL as they shift with the applied potential.

**Figure 7:**
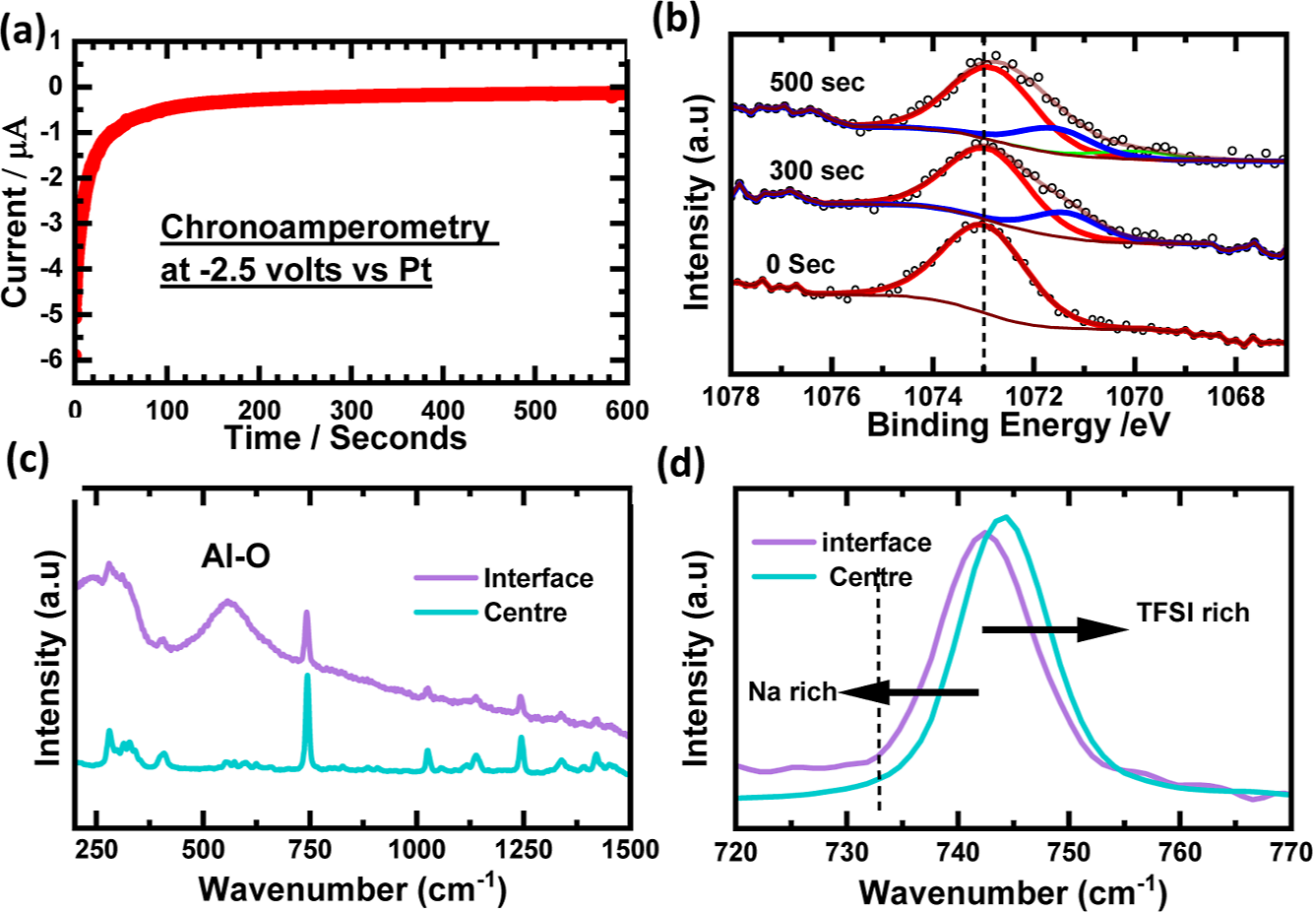
(a) Chronoamperometry data at −2.5 V vs Pt for Na deposition. (b) Na 1s spectra at different intervals of time at the electrode–electrolyte interface. (c,d) Raman spectra acquired at different points at the electrode–electrolyte interface and the center of the electrochemical cell.

To further investigate the behavior of Na species on the Al electrode, chronoamperometry was carried out at a potential of −2.5 V, as shown in Figure 7. At the interface, the Na 1s peak is broadened toward the lower BE, which supports the reduction of Na TFSI. To complement the XPS results, we performed operando Raman spectroscopy at precisely the same point of the electrode–electrolyte interface. The current study focused on monitoring the Na 1s behavior and the Raman signal during a single chronoamperometry measurement, although future investigations could include monitoring additional core levels. For this, an integrated Raman spectrometer was used. The Raman spectroscopy could be carried out in the same workflow and on the same spot on the sample that is being used for XPS analysis. Raman spectroscopy is routinely used to obtain the molecular fingerprint of constituent materials. Raman spectroscopy can also provide information on the ionic interactions of constituent anions and cations in the IL media.^43^ A reference spectrum was acquired at the center of the basin without any applied bias. In the spectra of BMIM TFSI, the Raman peak around 745 cm^–1^ is dependent on the presence of free or coordinated anions. With the addition of NaTFSI, the population of free anion decreases due to the formation of [Na (TFSI)_2_]^−^ clusters. This phenomenon causes broadening and a blue shift of this Raman peak.^44^ However, during chronoamperometry, the 745 cm^–1^ mode undergoes a red shift. The shift is consistent along the interface. This shift could be due to the reduced concentration of TFSI anions, making the interface region more Na-rich.

**Figure 8:**
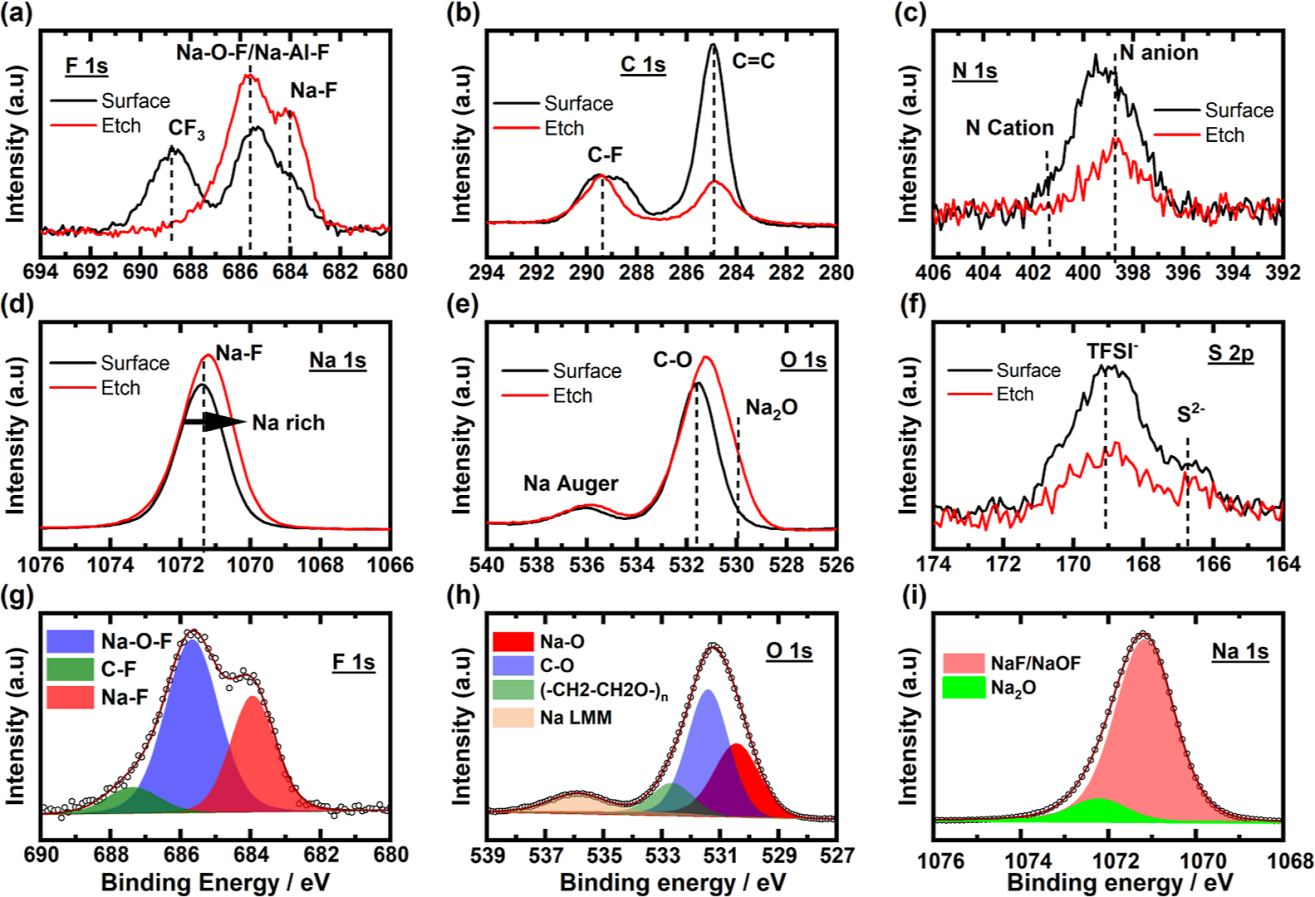
Ex situ XPS analysis of the Al electrode after cycling (a–f) surface scan and subsurface scan after 30 s Ar^+^ etching; (g–i) fitted XPS data of the etched surface.

To further investigate the nature of the Na species on the surface of the WE, chronoamperometry was carried out externally at −2.5 V. After chronoamperometry, the aluminum electrode was taken out, washed with acetone, and then transferred to the XPS for further analysis. It is to be noted that this process will inadvertently introduce surface oxidation, impacting the surface chemistry of the SEI layer. However, the presence of a hydrophobic IL on the surface could decelerate oxygen permeation into the bulk. The chemical information obtained through this ex situ measurement is meant to validate the formation of Na-rich SEI formed through the reduction of NaTFSI. After transferring the electrode for XPS measurements, a clear deposition was observed below the interface and can be seen on the optical image of the Al electrode (Figure S4). Two sets of spectra were acquired, first at the surface and second on the subsurface, after 60 s of Ar ion etching (2000 eV), as shown in Figure 8. Significant differences could be observed in the spectra of fluorine and carbon, Figure 8a,b, respectively. The relative intensities of carbon, nitrogen, and sulfur decreased with etching, while those of fluorine, sodium, and oxygen increased. This indicates that the surface is still covered with IL, which is removed after the etching.

The etched surface, SEI, is rich in Na, F, and O. In the fluorine spectra, the region between 688 and 690 eV corresponds to the CF_3_ groups. Since sulfur and CF_3_ are from the TFSI anion, the decrease in their peak intensities indicates that the SEI is formed mostly from the decomposition of the BMIM cation. Previous studies on IL-based electrolytes have shown that NaF and Na_2_O dominate the composition of the SEI. The same is evident here as well. Looking at the fitted F 1s, O 1s, and Na 1s spectra, fluoride and oxyfluoride are the dominant species here (Figure 8g–i). The F 1s spectra consist of two distinct peaks at 683.9 ± 0.2 eV and 685.7 ± 0.2 eV, corresponding to Na–F and oxyfluoride (Na–O–F) chemical environments, respectively.^45^ A relatively small contribution of the C–F environment could also be seen at 687.4 ± 0.2 eV. The sodium spectra are dominated by the Na–F peak at 1071.1 ± 0.2 eV, while a contribution of the Na–O environment at 1072.2 ± 0.2 eV could also be seen.^46^ However, the increased intensity of the Na can be related to an increased concentration of Na in the bulk. This matches well with Na 1s operando spectra taken during chronoamperometry (Figure 7b), where an increased concentration of Na species was observed with time. The presence of hydrophobic IL on the surface saves the deposited SEI from progressive oxidation into the bulk. The O 1s spectra consist of three peaks at 530.4 ± 0.2, 531.4 ± 0.2, and 532.7 ± 0.2 eV, corresponding to Na–O, C–O, and polymeric C–H–O environments, respectively. The higher energy peak at 535.9 ± 0.2 eV corresponds to a component of the Na KL_1_L_23_ Auger feature. The results obtained from the ex situ measurements suggest that the SEI becomes sodium-rich due to the reduction of the NaTFSI salt. The deposition of a Na-rich SEI was further corroborated with SEM and energy dispersive X-ray spectroscopy (EDS) on the electrode (Figure S4). Considering the bulk sensitivity of EDS, the results support the presence of sodium and sulfur in bulk, as seen from the depth profile.

## 5 Conclusions

This work extends the current analytical capability of a standard X-ray photoelectron spectrometer to directly probe the electrochemical processes at the electrode–electrolyte interfaces under operando conditions. Here, we integrated an electrochemical cell with a photoelectron spectrometer equipped with independent bias inputs. With the use of an IL as the electrolyte, we have performed operando electrochemistry inside the UHV XPS analysis chamber using a 3-electrode cell. By optimizing the speed of the electrochemical measurement with the acquisition speed of the photoemission spectra, true operando electrochemical XPS measurements can be performed. By using a CV scan rate of 2 mVs^–1^ and fast snapshot XPS spectra collected at 10 s per core level, it is possible to integrate the two techniques. As a proof of concept, we studied the behavior of Na ions in [BMIM][TFSI] at an Al WE during CV measurements.

By focusing XPS analysis at the interface of the WE and IL electrolyte, the behavior of species at the electrode surface and the bulk electrolyte were analyzed due to the applied potential. Species in contact with the grounded WE experienced a negligible shift in comparison to a ∼−1.0 eV/V shift observed in bulk IL components, which facilitates the identification of species located at the surface that are involved in the formation and evolution of the SEI. We observed changes in the Na 1s and F 1s spectra, corresponding to these processes occurring at the WE. We also demonstrated the integration of Raman spectroscopy as a complementary technique to understand the molecular dynamics of the electrode–electrolyte interface. Both the Raman spectroscopy and XPS spectroscopy identify a Na-rich environment at the surface of the Al electrode, which was confirmed by ex situ analysis of the cycled electrode. This work opens a promising route to operando study the interfacial charge transfer dynamics of electrochemical processes related to catalysis, corrosion, supercapacitor, optoelectronics, and many more. Further developments in instrumentation and data interpretation are required to reduce the time and improve the energy resolution of the measurement to incorporate faster chemical processes.

## Supporting Information Available

UPS spectra of an IL showing a shift in the valence band edge;operando interface spectra of Al 2p, C 1s, F 1s, and Na 1s core levels acquired during CV within the potential windows of 0 to −2.5 V;and SEM and EDS images of the deposited Na SEI on the aluminum electrode (PDF)
