# Supplementary material for: Monitoring the Behavior of Na Ions and Solid Electrolyte Interphase Formation at an Aluminum/Ionic Liquid Electrode/Electrolyte Interface via Operando Electrochemical X-ray Photoelectron Spectroscopy
Source: ACS Appl Mater Interfaces. 2024 Jun 27;16(27):35675–85. doi: 10.1021/acsami.4c02241 (PMC11247424; doi:10.1021/acsami.4c02241)
Supplement: Supplementary file 1 — am4c02241_si_001.pdf [file am4c02241_si_001.pdf]

Supporting Information

Monitoring the behaviour of Na ions and SEI Formation at an Aluminium/Ionic liquid electrode/electrolyte interface via Operando Electrochemical XPS

Roxy Lee[1], Tim S. Nunney[2], Mark Isaacs[1,3], Robert Palgrave[1], Avishek Dey[1,4,*]

[1]Department of Chemistry, University College London, 20 Gordon Street, London, WC1H 0AJ, United Kingdom

[2]Thermo Fisher Scientific, Unit 1, The Felbridge Centre, East Grinstead, West Sussex, RH19 1XP, United Kingdom

[3]HarwellXPS, Research Complex at Harwell, Rutherford Appleton Lab, Didcot OX11 0FA, United Kingdom

[4]The Faraday Institution, Quad One, Harwell Science and Innovation Campus, OX11 0RA, Didcot, UK

Corresponding author: A.Dey@ucl.ac.uk

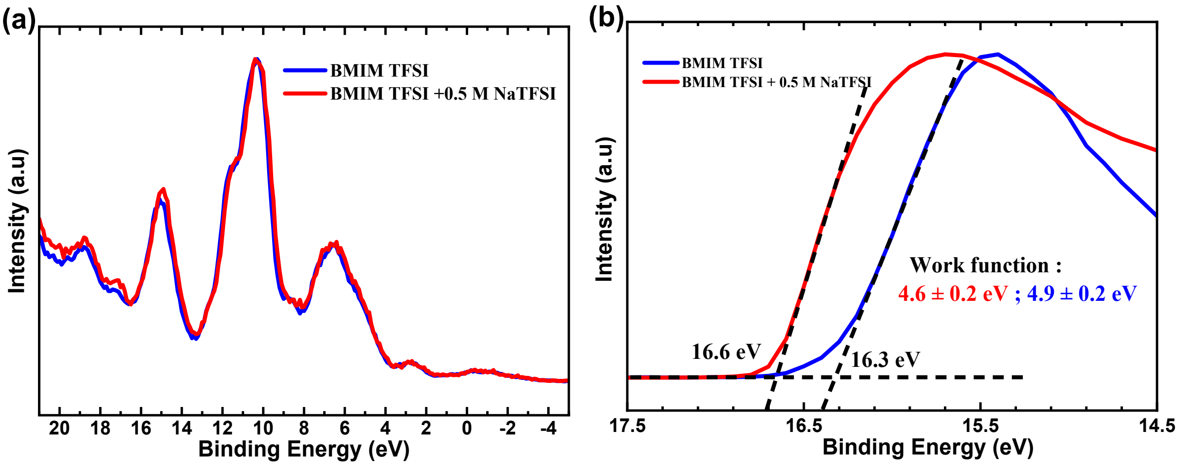

Figure S1. (a) He 2 (44 eV) UPS spectra of BMIM TFSI and BMIM TFSI+0.5m NaTFSI , (b) He 1 (21.2 eV) UPS spectra of the ionic liquid and electrolyte to measure the corresponding work functions.

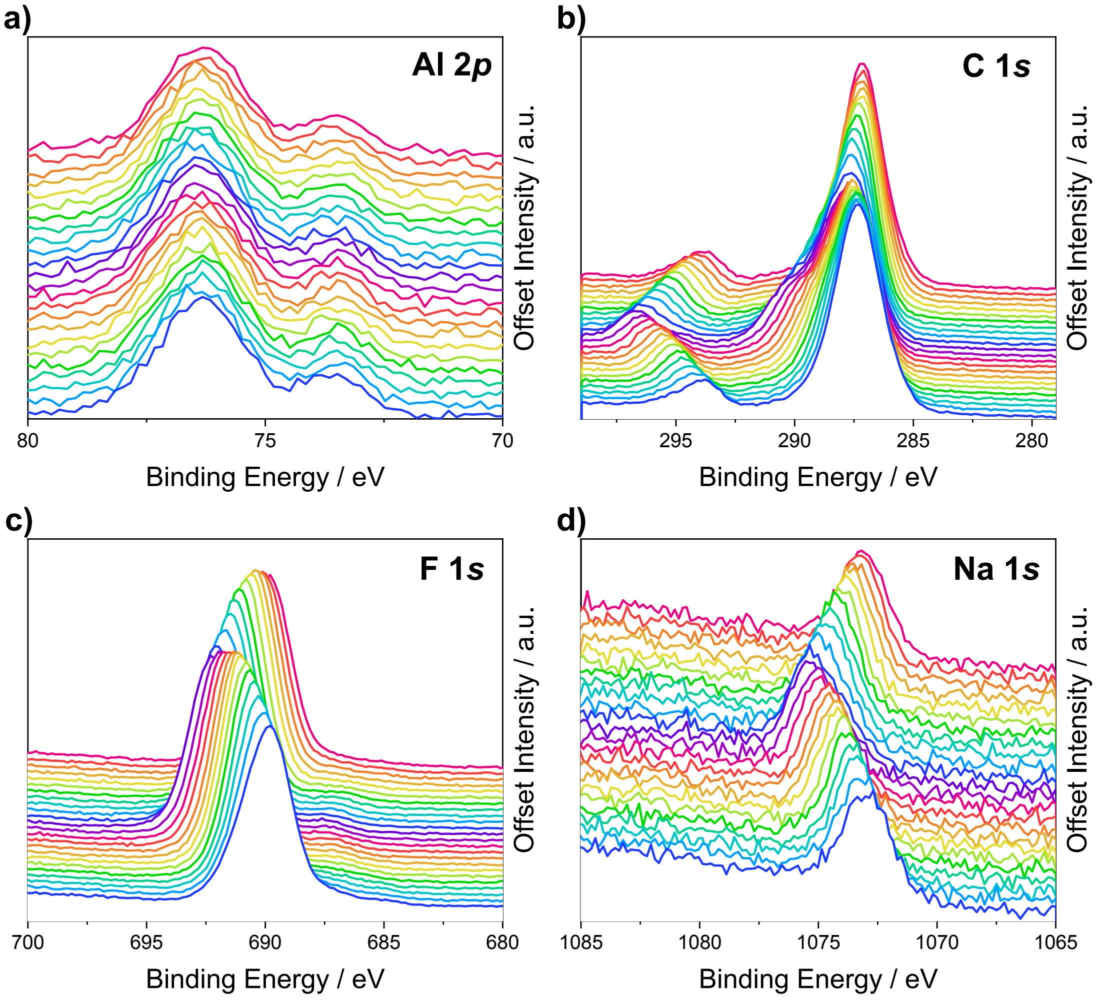

Figure S2. Operando XPS spectra taken at the WE/IL interface during CV measurement from 0 to -2.5 V.

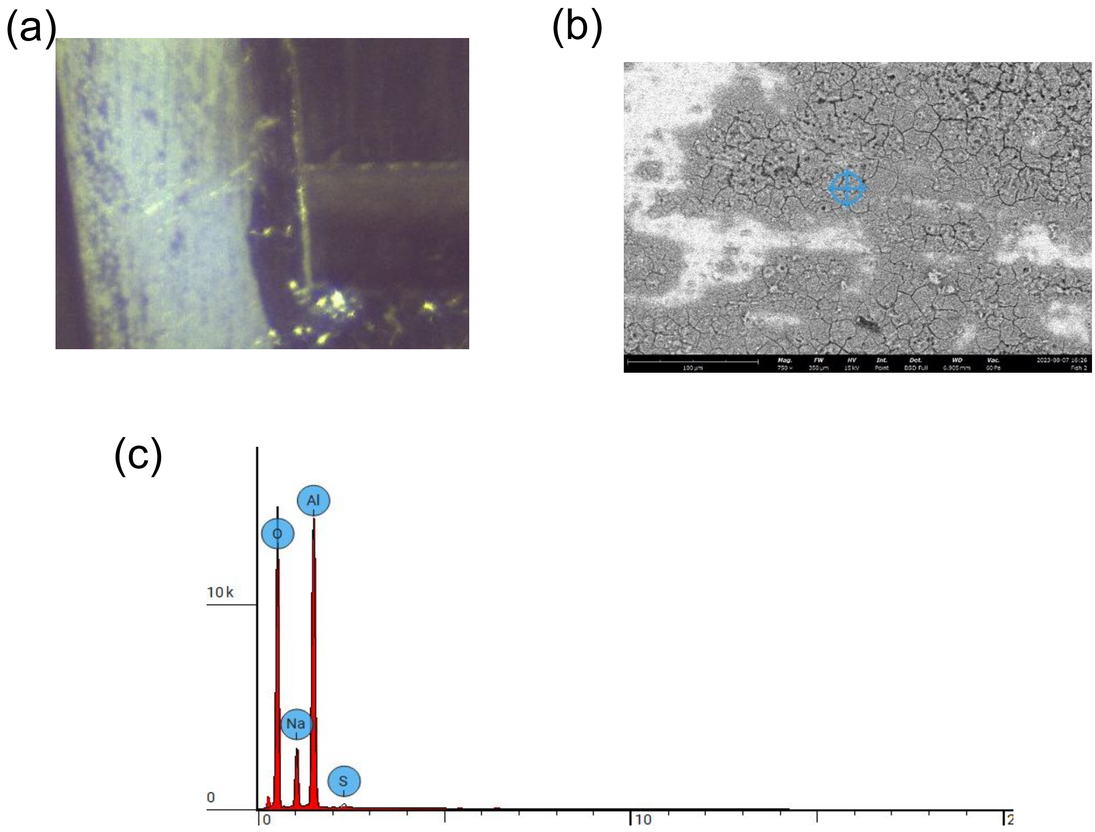

Figure S3. (a) Optical Image of the Al electrode after deposition of sodium species. The bright region corresponds the deposited sodium. ,(b) SEM image of the Sodium film and (c) corresponding EDS map.
